# Genetic silencing of K_Ca_3.1 inhibits atherosclerosis in ApoE null mice

**DOI:** 10.1080/19336950.2025.2538864

**Published:** 2025-08-03

**Authors:** P. Alam, D. L. Tharp, H. J. Bowles, L. A. Grisanti, H. Bui, S. B. Bender, D. K. Bowles

**Affiliations:** a Pathology and Integrative Biomedical Sciences, University of Missouri, Columbia, MO, USA; b Dalton Cardiovascular Research Center, University of Missouri, Columbia, MO, USA; c NextGen Precision Health, University of Missouri, Columbia, MO, USA; d Research, Harry S. Truman Memorial Veterans Hospital, Columbia, MO, USA

**Keywords:** K_Ca_3.1, gene silencing, brachiocephalic artery, atherosclerosis, macrophage, PPAR signaling, mitochondrial function

## Abstract

Increased expression of K_Ca_3.1 has been found in vascular smooth muscle cells (SMC), macrophages, and T cells in atherosclerotic lesions from humans and mice. Pharmacological inhibition of K_Ca_3.1 in limiting atherosclerosis has been demonstrated in mice and pigs, however direct, loss-of-function, i.e. gene silencing, studies are absent. Therefore, we generated K_Ca_3.1^−/−^Apoe^−/−^ (DKO) mice and assessed lesion development in the brachiocephalic artery (BCA) of DKO versus Apoe^−/−^ mice on a Western diet for 3 months. In BCAs of DKO mice, lesion size and relative stenosis were reduced by ~70% compared to Apoe^−/−^ mice, with no effect on medial or lumen area. Additionally, DKO mice exhibited a significant reduction in macrophage content within plaques compared to Apoe^−/−^ mice, independent of sex. *In vitro* migration assays showed a significant reduction in migration of bone marrow-derived macrophages (BMDMs) from DKO mice compared to those from Apoe^−/−^ mice. *In vitro* experiments using rat aortic smooth muscle cells revealed inhibition of PDGF-BB-induced MCP1/Ccl2 expression upon K_Ca_3.1 inhibition, while activation of K_Ca_3.1 further enhanced MCP1/Ccl2 expression. Both *in vivo* and *in vitro* analyses showed that silencing K_Ca_3.1 had no significant effect on the collagen content of plaque. RNAseq analysis of BCA samples from DKO and Apoe^−/−^ mice revealed PPAR-dependent signaling as a potential key mediator of the reduction in atherosclerosis due to K_Ca_3.1 silencing. Overall, this study provides the first genetic evidence that K_Ca_3.1 is a critical regulator of atherosclerotic lesion development and composition and provides novel mechanistic insight into the link between K_Ca_3.1 and atherosclerosis.

## Introduction

Despite lipid-lowering and emerging anti-inflammatory agents, atherosclerosis remains the leading cause of death in both men and women in the United States [1,2]. Over 20 million Americans >20 years of age have coronary heart disease (CHD), and each year ~635,000 Americans have a new coronary attack, and ~300,000 have a recurrent attack [3]. Atherosclerosis is a chronic, inflammatory, and proliferative disease that develops over decades, involving multiple cell types, including endothelial cells, smooth muscle cells (SMCs), fibroblasts, macrophages, T-cells, B-cells, and platelets. The intermediate-conductance Ca^2+^ -activated K^+^ channel (K_Ca_3.1) is expressed in all of these cell types and plays a crucial role in T-cell, B-cell, fibroblast, and SMC proliferation, as well as the migration of SMCs, macrophages, and platelet coagulation [4–8], leading to the consideration of K_Ca_3.1 modulators as potential therapies for vascular disease [9,10].

Increased expression of K_Ca_3.1 has been found in atherosclerotic lesions from humans and mice [7,11] and several studies have shown systemic delivery of K_Ca_3.1 inhibitors can attenuate atherosclerosis lesion development in mice [7,11–13]. Synthetic, proliferating SMC increases the expression of K_Ca_3.1, such that it becomes the dominant K^+^ channel [8,14]. Although the cell type-specific relative contribution of K_Ca_3.1 activation during atherosclerosis has not been determined, its upregulation has been observed in neointimal SMCs in balloon-injured rat carotid arteries [7,15] as well as atherosclerotic lesions in mice and humans [7]. In addition, we have shown that acute administration of the K_Ca_3.1 inhibitor, TRAM-34, during coronary angioplasty in a swine model of coronary restenosis can inhibit lesion development, predominantly by targeting SMC proliferation [5].

Migration of SMC from the media to the intima is a major contributor to both restenosis and atherosclerosis, and we [5,8] and others [6,7] have shown that K_Ca_3.1 is critical for SMC migration. Together, these studies support the hypothesis that the upregulation of K_Ca_3.1 is a major contributor to SMC migration and proliferation during atherosclerosis and restenosis. In addition to the potential effects on SMC, K_Ca_3.1 inhibitors [7,11] reduce lesion macrophage content in Apoe^−/−^ mice. In macrophages, K_Ca_3.1 regulates migration, M1/M2 polarization, respiratory burst, and pathogen killing [16]. Pharmacological blockade of K_Ca_3.1 inhibits M1 polarization and increases the M2/M1 ratio in advanced plaques [11]. In addition to the direct role of macrophage K_Ca_3.1 activation, K_Ca_3.1 may also regulate interactions between SMC and macrophages by modulating the inflammatory phenotype of SMCs [10]. The “inflammatory state” of SMCs in the lesion cap is crucial, as these cells secrete chemokines such as MCP1/Ccl21 [17], which recruit monocytes/macrophages that degrade the fibrous cap and contribute to the plaque instability [18,19].

In summary, while there is substantial evidence supporting the role of K_Ca_3.1 in SMC and macrophage function in atherosclerosis, the evidence to date is derived from *in vitro* studies on selected cell types or *in vivo* pharmacological investigations. The purpose of the current study was to provide the first genetic silencing of K_Ca_3.1 in the context of atherosclerosis development *in vivo*. Results of this study have been available as a pre-print [20]

## Material and methods

### Ethics statement

Experimental protocols performed adhered to ARRIVE guidelines, were approved by the University of Missouri Animal Care and Use Committee (Protocol #42761) and conducted in accordance with the “Principles for the Utilization and Care of Vertebrate Animals used in testing, Research and Training.” Animals were anesthetized with Ketamine/Xylazine and euthanized by exsanguination and removal of the heart in accordance with American Veterinary Medical Association guidelines. Detailed methods can be found in Supplement.

### Statistical analysis

All data are presented as mean ± SE. Statistical analyses were performed with the Prism 10 statistical software (GraphPad Software, San Diego, CA). Before comparisons, normality was assessed using the Shapiro–Wilk method, and variance homogeneity was determined using F testing. Normal distributions with homogeneous variances were compared using unpaired, two-tailed Student t tests, one-way or two-way ANOVA as appropriate. A *p* value threshold of 0.05 was pre-established to designate statistical significance. Statistical cutoffs for differentially expressed genes were − 1.0 ≤ log^2^FC ≥ 1.0 change in expression with an adjusted *p* < 0.05.

## Results

### Apoe^−/−^Kcnn4^−/−^ (DKO) mice

**Figure 1.**
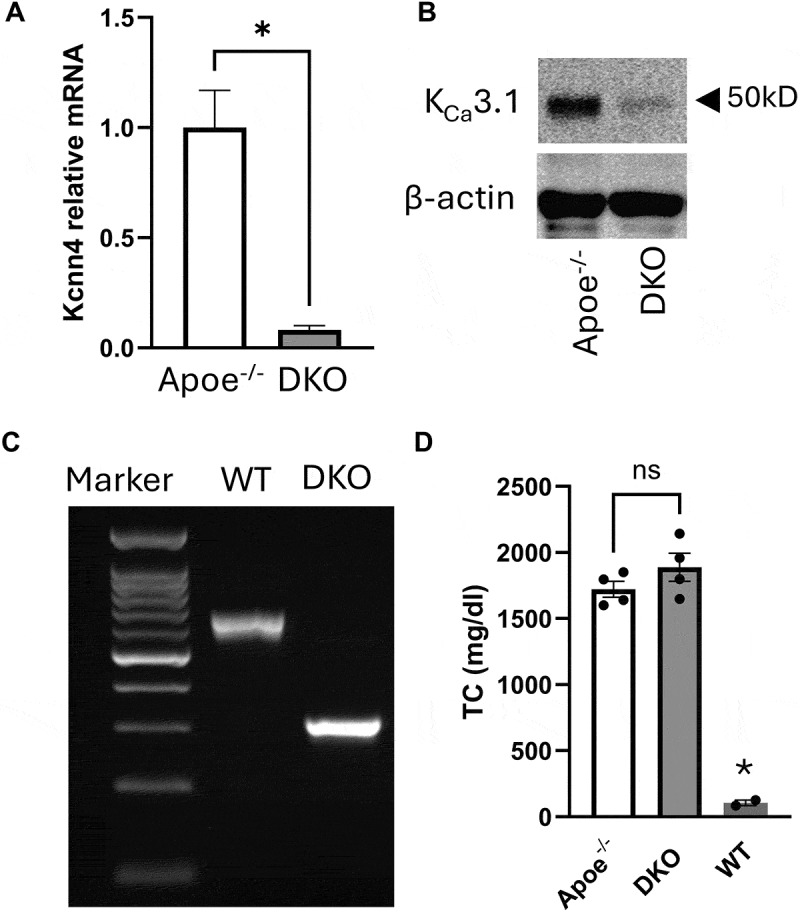
K_Ca_3.1 silencing in DKO mouse. Loss of K_Ca_3.1 mRNA (A) and protein (B) in DKO compared to Apoe^−/−^, (C) PCR gel of Apoe showing 500 bp deletion in DKO compared to WT, and (D) producing similar elevated levels of total plasma cholesterol in DKO (*n* = 4) and Apoe^−/−^ (*n* = 4) vs. WT (*n* = 2). *p < 0.05. *ns* = non significant.

In DKO mice, we confirmed a significant reduction in K_Ca_3.1 mRNA (Figure 1(A)) and protein (Figure 1(B)) expression compared to Apoe^−/−^ mice. Genotyping of the Apoe allele showed a 500 bp deletion in the DKO mice, when compared to WT mice (Figure 1(C)). Founder DKO mice (*n* = 4) also demonstrated a similar increase in plasma cholesterol levels as Apoe^−/−^ mice (*n* = 4) compared to wild-type (WT, *n* = 2) controls. Importantly, no significant difference was observed in cholesterol levels between DKO and Apoe^−/−^ mice (Figure 1(D)).

### Group characteristics

Group comparisons for body weight (BW) and total cholesterol are shown in Table 1. Overall, male mice were heavier than females across genotypes. Male Apoe^−/−^ and DKO body weights were similar, while DKO female mice were heavier than Apoe^−/−^ females. While total cholesterol was lower in Apoe^−/−^ females compared to Apoe^−/−^ males, K_Ca_3.1 silencing had no effect within either sex.

**Table 1.** Group characteristics.

	Male		Female	
	Apoe^−/−^	DKO	Apoe^−/−^	DKO
BW (g)	35.4	37.2	25.8^#^	29.8^*#^
SE	±1.3	±1.4	±0.7	±0.9
N	12	12	12	9
TC (mg/dl)	1670	1706	1303^#^	1521
SE	±76	±124	±64	±75
N	12	12	12	9

*p < 0.05 vs. Apoe^−/−^; ^#^vs. male.

### Brachiocephalic artery morphometry

**Figure 2.**
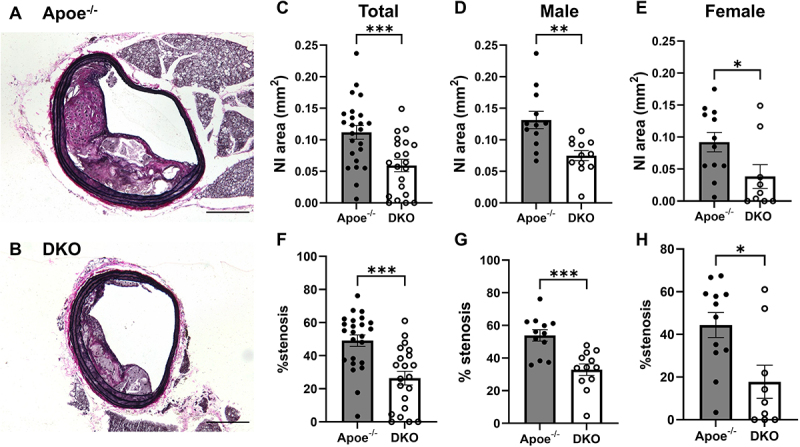
K_Ca_3.1 silencing reduces atherosclerotic plaque size. Representative VVG stained BCA sections Apoe^−/−^ (A) and DKO (B) mice. Scale bar = 200 µm. Morphometric data showing (C) neointimal (NI) areas (total), (D) NI in males, (E) NI in females, and (F) % stenosis in Apoe^−/−^ and DKO (total), (G) % stenosis in Apoe^−/−^ and DKO males, and (H) % stenosis in Apoe^−/−^ and DKO females. While females had overall smaller NI and % stenosis vs. males in both groups, silencing K_Ca_3.1 significantly reduced both NI area and % stenosis in both sexes. Group data represent Apoe^−/−^ *n* = 24 total; *n* = 12 male, 12 female, and DKO *n* = 21 total; *n* = 12 male and 9 female; *p < 0.05, **p < 0.01, ***p < 0.001.

We examined atherosclerosis development in the brachiocephalic artery (BCA). As reported previously [21], Apoe^−/−^ mice fed a Western diet developed complex atherosclerotic lesions consisting of a fibrous, cellular matrix overlying lipid cores (Figure 2(A)). Although both neointimal area and relative stenosis were lower in female compared to male mice in both Apo^−/−^ and DKO mice, DKO mice demonstrated significantly reduced neointimal size (NI; Figure 2(A,C–E)) and relative stenosis (Figure 2(B,F–H)) in both sexes.

### Silencing K_Ca_3.1 alters atherosclerotic plaque composition

**Figure 3.**
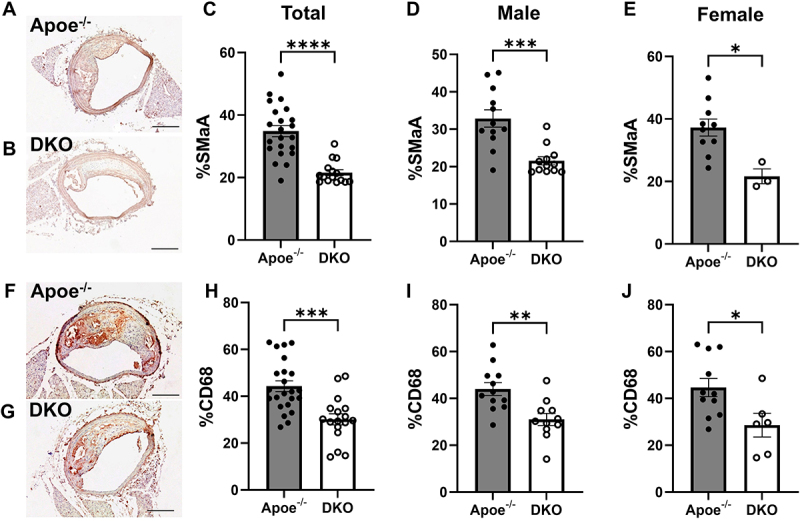
K_Ca_3.1 silencing reduces intimal smooth muscle and macrophage content. Representative BCA sections from Apoe^−/−^ (A) and DKO (B) mice probed with anti-smooth muscle alpha actin (SMαA) and (C) group data for relative SMαA positive intimal area (total), as well as in males (D), and in females (E). Data presented as mean ± S.E, Apoe^−/−^ *n* = 22 total; *n* = 12 male, 10 female, and DKO *n* = 15 total; *n* = 12 male and 3 female; *p < 0.05, ***p < 0.001, ****p < 0.0001. Representative BCA sections from Apoe^−/−^ (F) and DKO (G) mice probed with CD68 and (H) group data for relative CD68 positive intimal area (total), as well as for males (I), and females (J). Data presented as mean ± S.E, Apoe^−/−^ *n* = 23 total; *n* = 12 male, 11 female, and DKO *n* = 17 total; *n* = 11 male and 6 female; *p < 0.05, **p < 0.01, ***p < 0.001.

**Figure 4.**
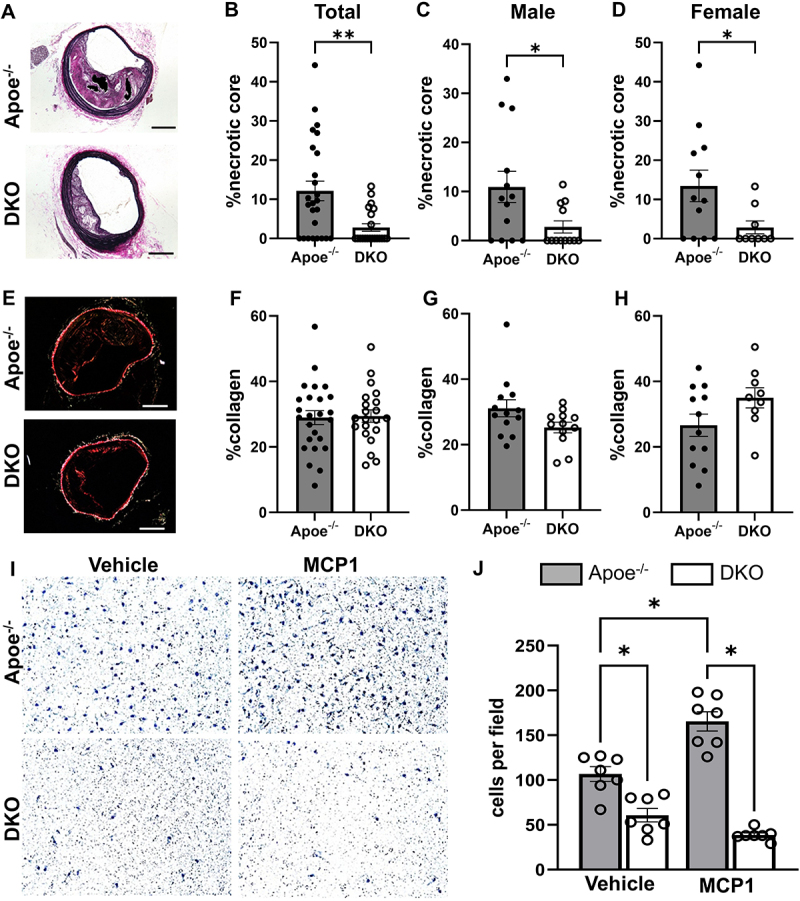
K_Ca_3.1 silencing reduces necrotic core size with no effect on relative intimal collagen content and inhibition of macrophage migration. (A) Representative VVG stained BCA sections from Apoe^−/−^ and DKO analyzed for necrotic core as defined in methods. (B-D) group data for relative necrotic core area. (E) Representative BCA sections from Apoe^−/−^ and DKO mice stained with picrosirius red under polarized light and (F-H) group data for relative positive intimal area. Data presented as mean ± S.E, Apoe^−/−^ *n* = 25 total; *n* = 13 male, 12 female, and DKO *n* = 21 total; *n* = 12 male and 9 female; *p < 0.05, **p < 0.01. (I) Representative membranes from chemotaxis chambers showing migrated BMDM cells from Apoe^−/−^ and DKO in response to vehicle or MCP1/Ccl2 stimulation. (J) quantified group data presented as mean ± S.E *n* = 7 per group, *p < 0.05.

We and others have previously shown that inhibiting K_Ca_3.1 reduces smooth muscle cell proliferation and migration and macrophage activation and migration [7,8,11]. Consistent with these findings, both SMC (Figure 3A–E) and macrophage (Figure 3(F–J)) content were significantly reduced in lesions from DKO mice when compared to those from Apoe^−/−^ mice, in both sexes. It has been shown that the majority of SMCs within atherosclerotic lesions are of medial origin and migrate into the intima during lesion development [22–24]. Previous studies by us and others [7,8,25] have shown that the K_Ca_3.1 inhibitor, TRAM-34, inhibits SMC migration *in vitro* and reduces SMC content in atherosclerotic plaques. The effect of K_Ca_3.1 silencing in reducing SMC content within the lesion suggests that K_Ca_3.1 activation is involved in medial-to-intimal SMC migration in atherosclerosis development. Additionally, our results show a significant reduction in the necrotic core size in DKO mice compared to Apoe^−/−^ mice (Figure 4(A,B)), in both sexes (Figure 4(C,D)). In contrast, K_Ca_3.1 silencing, and sex had no significant effect on the relative collagen content in the lesions (Figure 4(E–H)). Overall, lesions in DKO animals were smaller, with reduced smooth muscle and macrophage content, and a dramatically diminished necrotic core. Together, these findings suggest that K_Ca_3.1 silencing leads to smaller lesions with features indicative of a more stable plaque.

We performed *in vitro* studies to investigate potential mechanisms of reduced macrophage content in atherosclerotic lesions after K_Ca_3.1 silencing. Migration of BMDM from DKO mice was significantly reduced compared to Apoe^−/−^ in both male and female mice (Figure 4(I,J)), providing direct genetic evidence supporting previous findings demonstrating a role for K_Ca_3.1 in macrophage activation and infiltration [7,11,26]. In addition, given the potential for intimal SMCs to contribute to macrophage infiltration via expression of chemotactic factors, such as MCP1/Ccl2, we examined the role of K_Ca_3.1 in MCP1/Ccl2 expression in SMC (Supplementary Figure S1). In rat aortic smooth muscle cells (RASMCs), K_Ca_3.1 activity was modulated using specific siRNA (siKcnn4), along with its pharmacological inhibition by TRAM-34 and activation by SKA-31. As previously, we observed a significant increase in K_Ca_3.1 expression after PDGF-BB treatment [8], which was significantly inhibited following K_Ca_3.1 inhibition by either siKcnn4 or TRAM-34, and augmented by SKA-31 (Supplementary Figure S1A-C), confirming a positive feedback role of K_Ca_3.1 on self-expression [5,8]. Similarly, PDGF-BB-induced MCP1/Ccl2 expression was inhibited following K_Ca_3.1 inhibition by either siKcnn4 or TRAM-34 and augmented by SKA-31 (Supplementary Figure S1D-F). Furthermore, using rat aortic endothelial cells (RAOECs), we observed no effect of K_Ca_3.1 inhibition, either by siRNA (Supplementary Figure S2A) or the inhibitor TRAM34 (Supplementary Figure S2B), nor its activation by SKA31 (Supplementary Figure S2C), on the expression of MCP1/Ccl2. This suggests that the observed changes in MCP1/Ccl2 expression are likely derived from SMCs. In contrast, no significant effect was observed on Col1a1 expression following either PDGF-BB treatment or inhibition of K_Ca_3.1 (Supplementary Figure S3A, B); however, we did observe a main effect of K_Ca_3.1 activation by SKA-31 (Supplementary Figure S3C). Thus, these studies confirm a putative multicellular contribution of K_Ca_3.1 to the macrophage content of atherosclerotic lesions.

### Putative beneficial transcriptome changes in atherosclerotic lesions with K_Ca_3.1 silencing

**Figure 5.**
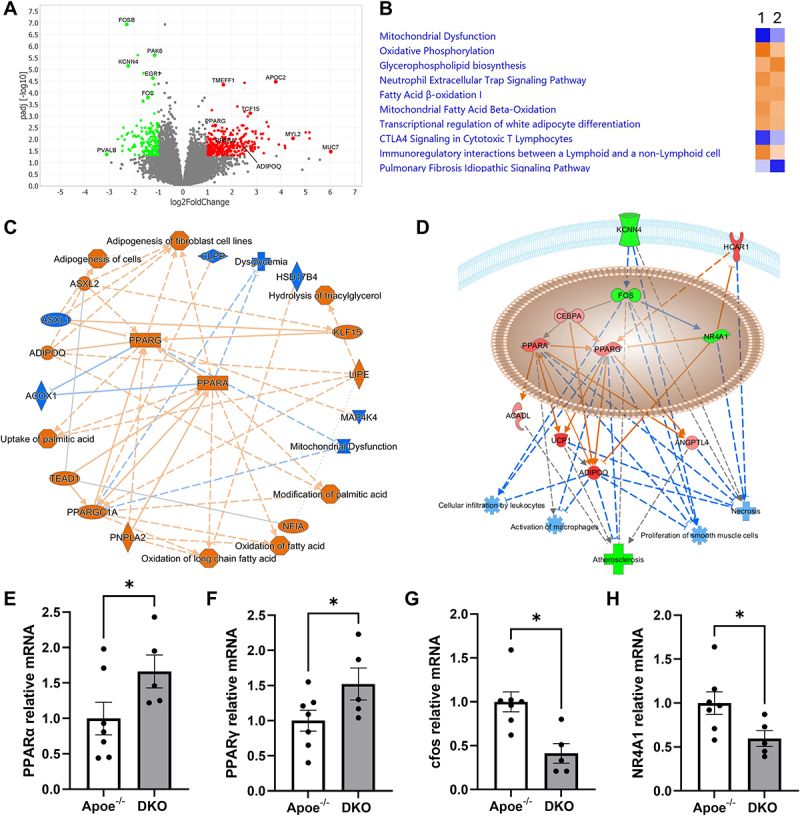
Transcriptomic responses in BCA affected by silencing K_Ca_3.1. (A) volcano plot of differentially expressed genes in BCA from DKO vs Apoe^−/−^ mice. Upregulated genes coded red and downregulated genes coded green (log [2]fold change < −1 and > 1, adjusted *p* value < 0.05). (B) comparison of top 10 pathways affected in DKO vs Apoe^−/−^ (1) vs. rosiglitazone treatment effect on bone marrow mesenchymal stem cells (GSE10192.GPL1261.test6; 2). (C) graphical summary of effects of K_Ca_3.1 silencing in atherosclerosis as generated by IPA showing the central role of PPAR activation. (D) downstream regulated biological outcomes based on major DEG genes with K_Ca_3.1 silencing leading to putative beneficial outcomes related to reduced atherosclerosis, including inhibition of cellular infiltration of leukocytes, reduced activation of macrophages, reduced proliferation of SMCs, and reduced necrosis. Green indicated downregulated genes, red indicates upregulated genes, blue indicates predicted inhibition. Key model genes in 5D were validated with qRT-PCR in aorta showing significant increases in PPARα (E), PPARγ (F), and decreased cfos (G) and NR4A1(H). Group data presented as mean ± S.E for Apoe^−/−^ (n = 7) and DKO (n = 5), *p < 0.05.

In order to explore potential novel mechanisms underlying the beneficial impact of K_Ca_3.1 silencing, we performed bulk RNA sequencing of diseased BCA from both Apoe^−/−^ and DKO mice to identify unique transcriptomic signatures induced by K_Ca_3.1 silencing in atherosclerosis. Analysis identified 449 differentially expressed genes (DEGs) that significantly altered (152 downregulated and 297 upregulated, Figure 5(A)). DEGs were used to identify key pathways using gene ontology (GO) enrichment analysis and directional gene changes in IPA. The GO analysis using Metascape (https://metascape.org) revealed significant activation of the respiratory electron transport chain, PPAR signaling pathways and metabolic pathways (Supplementary Figure S4A). Moreover, regulatory factor analysis demonstrated a predominant role of PPARs (Supplementary Figure S4B). Similarly, IPA identified the top four statistically enriched pathways as mitochondrial, i.e. decreased mitochondrial dysfunction, increased respiratory electron transport, increased oxidative phosphorylation, and increased mitochondrial fatty acid oxidation (Supplementary Figure S4A). Comparison analysis within IPA aligned DKO with great similarity to the top enriched pathways identified from transcriptomic analysis of rosiglitazone treatment (Figure 5(B)). DEGs within the PPAR signaling pathway included significant upregulation of PPARα, PPARγ, and PPARGC1A (Figure 5(C)). Accordingly, IPA identified the PPAR agonist, rosiglitazone, as the top upstream regulator activated with 45 of 52 predicted downstream genes activated (Supplementary Table S1). Overall, transcriptomic analysis identified PPAR pathway activation as a top putative mechanism underlying the effects of K_Ca_3.1 silencing on atherosclerosis. Examination of top DEG pathways downstream of K_Ca_3.1 inhibition with cellular processes known to be involved in atherosclerosis reveals a novel model of the mechanisms underlying K_Ca_3.1 silencing inhibiting atherosclerotic lesion development and altering plaque composition (Figure 5(D)). K_Ca_3.1 silencing leads to alterations in key nuclear factors, including downregulation of cfos and NR4A1 and upregulation of PPARα, PPARγ, and PGC1α (1.8-fold, *p* < 0.05) subsequently predicted to lead to inhibition of key atherogenic processes including leukocyte infiltration, macrophage activation, SMC proliferation, and necrosis, resulting in inhibition of atherosclerosis observed in the current experiment. The expression of PPAR genes identified through RNA-seq DEG analysis, along with *in silico* GO and IPA analysis, for their potential involvement in the mechanisms underlying K_Ca_3.1 knockout-mediated disease protection, was further validated by qRT-PCR (Figure 5(E–H)). Since total BCA samples were used to perform RNA-seq, aorta samples from the corresponding animals were used for validation analysis.

## Discussion

The current study is the first to use *in vivo* gene silencing to examine the role of K_Ca_3.1 on atherosclerosis progression. Using a Apoe^−/−^Kcnn4^−/−^ mouse, we were able to demonstrate a reduction in atherosclerotic plaque size, SMC, and macrophage content and decreased necrotic core due to silencing K_Ca_3.1. While these outcomes are consistent with previous studies using pharmacological inhibition of K_Ca_3.1 *in vivo* [5,7], this study provides the first genetic validation of the role of K_Ca_3.1 in regulating atherosclerosis. In addition, subsequent transcriptomic analysis revealed novel putative mechanisms of K_Ca_3.1 silencing of an improved mitochondrial function and a transcriptomic profile similar to that induced by PPAR activation, both of which have been implicated in reducing atherosclerosis.

Our finding of a reduction in SMC content in lesions is consistent with previous studies showing that TRAM-34, an inhibitor of K_Ca_3.1, inhibits smooth muscle cell proliferation and migration [7,8,25]. Accordingly, one would expect a reduction in medial-to-intimal smooth muscle migration and expansion and secondary reduced intimal fibrosis during lesion development with K_Ca_3.1 inhibition. On the contrary, we did not see a reduction in BCA lesion collagen content as previously seen with TRAM-34 [12] and other studies, which demonstrated K_Ca_3.1 inhibition reduces fibrosis [10,27–29]. Although the relative collagen content remained unchanged with K_Ca_3.1 silencing, there was a significant reduction in relative necrotic core size in lesions of both male and female DKO mice. Atherosclerotic plaques prone to rupture and subsequent luminal thrombosis are characterized by large necrotic areas, fibrous cap thinning, apoptosis surrounding the necrotic core, and high levels of inflammatory cytokines and matrix proteases [30–32]. These thin cap fibroatheromas (TCFA) contribute to major adverse cardiovascular events (MACE), including sudden death. Necrotic cores contain free and esterified cholesterol and dead or senescent macrophages and form due to insufficient efferocytosis of foam cells [33,34]. The significant reduction in necrotic core content of the lesions with K_Ca_3.1 silencing results in lesions with characteristics of a more stable plaque and, consequently, is expected to reduce plaque rupture risk [35,36]. Thus, our study demonstrates that K_Ca_3.1 inhibition not only decreases lesion size but alters plaque composition toward a more stable phenotype.

Moreover, silencing K_Ca_3.1 led to a reduced macrophage content in both male and female lesions as assessed by the macrophage marker, CD68 (Figure 3(F–J)). This is consistent with previous findings that K_Ca_3.1 inhibitors reduce lesion macrophage content in Apoe^−/−^ mice [7,11]. In macrophages, K_Ca_3.1 has been shown to regulate migration, M1/M2 polarization, respiratory burst, and pathogen killing [16]. Pharmacological blockade of K_Ca_3.1 inhibits differentiation toward the M1 phenotype and increases the M2/M1 ratio in advanced plaques [11]. Inflammation is a potent regulator of collagen deposition and fibrosis, both of which directly influence plaque stability and the severity of atherosclerosis [37]. Inflammatory mediators, such as angiotensin II (Ang II), promote macrophage polarization toward the pro-inflammatory M1 phenotype, which destabilizes plaques by producing enzymes like matrix metalloproteinases (MMPs) that degrade the fibrous cap, weakening plaque integrity [38–40]. In contrast, M2 macrophages, which are anti-inflammatory, help maintain plaque stability by promoting collagen deposition and limiting excessive inflammation, thereby stabilizing the plaque [41,42]. Furthermore, studies have demonstrated that K_Ca_3.1 inhibition reduces vascular inflammation [43] and may shift macrophage polarization toward the M2 phenotype, thereby contributing to the formation of smaller, more stable plaques [35,36,44,45]. Thus, loss of K_Ca_3.1 in macrophages due to global silencing may contribute to the observed reduction in macrophage infiltration into atherosclerotic lesions, thereby further promoting plaque stabilization.

Alternatively, the findings that both K_Ca_3.1 inhibitors [11] and gene silencing show reductions in lesion macrophage content could also indicate a role of SMC K_Ca_3.1 through MCP1/Ccl2 expression and/or SMC cell transdifferentiation to foam cells. The cytokine MCP1/Ccl2 is one of several chemokines that play a key role in monocyte recruitment to atherosclerotic plaques [46]. Polymorphisms in the MCP1/Ccl2 promoter, thought to increase MCP1/Ccl2 expression, have been associated with CAD, chronic stable angina, and myocardial infarction [46]. Using MCP1/Ccl2 receptor-deficient mice to examine atherosclerosis, it was demonstrated that, in the absence of the receptor for MCP1/Ccl2, CCR2, there was a substantial reduction in arterial lipid deposition [47] and diminished numbers of macrophages in the arterial wall [48]. We provide evidence that K_Ca_3.1 positively regulates MCP1/Ccl2 expression in SMC (Supplementary Figure S1D-F), but not endothelial cells (Supplementary Figure S2). In isolated SMC, inhibition of K_Ca_3.1 by siRNA or TRAM-34 inhibits MCP1/Ccl2 expression, while activation of K_Ca_3.1 with SKA-31 greatly enhances MCP1/Ccl2 expression. Thus, silencing K_Ca_3.1 in SMC *in vivo* may contribute to reduced macrophage recruitment into the intima.

In addition, *in vitro* cholesterol loading of SMCs reduces smooth muscle differentiation markers (e.g. SMαA, SMMHC) and increases macrophage marker expression (CD68, Mac-2) [49,50]. Allaverdian et al. [51] concluded that approximately 50% of foam cells in human coronary lesions are of SMC, not monocyte, origin. Similarly, data using SMC lineage tracking in mice have concluded that the majority of intimal foam cells (up to 80%) are of SM cell origin [52]. This SMC to foam cell transdifferentiation is thought to be associated with a down regulation of myocardin [53], which we have shown is mediated, in part, by K_Ca_3.1 [5,8,25]. Given these observations, the reduced macrophage/foam cell content in lesions following K_Ca_3.1 inhibition may be attributed to effects on SMCs, rather than macrophage infiltration. Thus, K_Ca_3.1 may regulate SMC plasticity and its potential to transition into foam cells, contributing to plaque composition and stability. However, to more precisely define the contributions of SMCs versus macrophages to foam cell formation *in vivo*, future studies utilizing cell-specific knockout and/or lineage tracing models will be essential. These approaches will help delineate the distinct roles of each cell type in lesion formation and foam cell accumulation, ultimately advancing our understanding of the molecular mechanisms driving atherosclerosis.

In addition to the potential mechanisms underlying the beneficial effects of K_Ca_3.1 inhibition on atherosclerosis, transcriptomic analysis revealed putative novel effector signaling pathways associated with K_Ca_3.1. Specifically, this analysis highlighted a significant predicted enhancement of mitochondrial function and the activation of PPAR signaling upon K_Ca_3.1 silencing. Ingenuity Pathway Analysis (IPA) showed a striking similarity between the top canonical pathways affected in BCAs following K_Ca_3.1 silencing and those influenced by the PPAR agonist, rosiglitazone (Figure 5B,C). These pathways included improved mitochondrial function, enhanced fatty acid oxidation, and the inhibition of fibrosis, all of which are known to contribute to atherosclerosis regression and plaque stabilization. Previous studies have shown that PPAR agonists reduce vascular inflammation, plaque size, and atherosclerosis progression [54–57], as well as lower in-stent NI volume in non-diabetic patients [58]. For example, rosiglitazone has been shown to decrease atherosclerotic plaque size by reducing lipid deposition and macrophage infiltration while also decreasing MCP1/CCL2 levels and increasing adiponectin in the aorta [54] all similar to effects observed in the current study. However, given that endothelial cells, vascular smooth muscle cells (SMCs), monocytes/macrophages, and T cells all express PPARα and PPARγ [59], it is difficult to pinpoint which specific cell types are primarily responsible for the beneficial effects observed. To address this, further cell-specific studies, including lineage tracing and knockout models, will be essential to delineate the exact cellular mechanisms underlying the improvements in plaque stability and regression associated with K_Ca_3.1 inhibition.

Increased mitochondrial respiration and reduced mitochondrial dysfunction have been consistently associated with decreased atherosclerotic progression [60–65]. In the aortas of Ob/Ob, LDLR^−/−^ mice, low levels of cytochrome oxidase were linked to increased plaque formation and elevated MCP1/Ccl2 levels [60]. Similarly, in pig coronary plaques, cytochrome oxidase I and 4I1 were found to be reduced in macrophages from complex Stary III plaques compared to less advanced Stary I plaques [60]. Hypercholesterolemia results in a coordinated down-regulation of mitochondrial genes. Moreover, these genes are tightly connected in co-expression network modules related to mitochondrial biogenesis and antioxidant responses, possibly regulated by ERR-α/PGC1-α [66]. These findings suggest that improved mitochondrial activity could contribute to the stabilization and regression of atherosclerotic plaques. The effects of K_Ca_3.1 inhibition on mitochondrial function observed in our study align with these previous findings. Our transcriptomic analysis predicts that silencing K_Ca_3.1 not only enhances mitochondrial function but also activates signaling pathways similar to those triggered by PPAR agonists, such as rosiglitazone. Notably, rosiglitazone has been shown to increase mitochondrial biogenesis and function in tissues like the brain [67] and adipose tissue [68], and these processes are known to be protective against atherosclerosis. The parallels between the effects of rosiglitazone and the transcriptomic changes induced by K_Ca_3.1 silencing suggest that activation of the PPAR pathway may play a central role in the beneficial effects of K_Ca_3.1 inhibition. Notably, K_Ca_3.1 silencing increased UCP1 in BCAs in the current study. UCP1 is activated by PPARα and PPARγ [69] has been shown to inhibit atherosclerosis by reducing vascular inflammation [70]. This connection further underscores the potential for targeting K_Ca_3.1 as a therapeutic strategy for atherosclerosis, as improving mitochondrial function and activating PPAR pathways could collectively reduce vascular inflammation, improve plaque stability, and limit disease progression. Additionally, the observed predicted effects on mitochondrial function and PPAR signaling provide novel mechanistic targets for how K_Ca_3.1 silencing may promote atherosclerosis regression and plaque stabilization.

In conclusion, our study provides further support for the important contribution of K_Ca_3.1 activation in the progression of atherosclerotic lesion development and composition and provides novel insights into the mechanisms of the beneficial effect of K_Ca_3.1 inhibition on atherosclerosis. While previous studies have utilized *in vitro*, siRNA gene silencing of K_Ca_3.1 in isolated cell lines, our study provides the first evidence of a protective role for K_Ca_3.1 knockout *in vivo* in a model of atherosclerosis progression of complex plaques, which we believe adds valuable insight to the overall understanding of lesion development and composition. Together, our *in vivo*, *in vitro,* and transcriptomic findings implicate a novel model of the mechanisms underlying K_Ca_3.1 silencing, inhibiting atherosclerotic lesion development and altering plaque composition (Figure 5D) involving key nuclear factors cfos, NR4A1, PPARα, PPARγ, and PGC1α leading to a reduction in plaque size and altered composition. Together with previous studies, these findings support the potential therapeutic application of pharmacological inhibition of K_Ca_3.1 in limiting the progression of atherosclerosis. However, there are limitations to the current study. As noted, increased expression of KCa3.1 has been observed in multiple cell types involved in atherosclerotic lesion progression including smooth muscle, macrophages, fibroblasts, and T cells. Studies to date, including the current study, have relied on global gene silencing or systemic pharmacological interventions *in vivo*. Thus, while the current study supports an overall permissive role for K_Ca_3.1 during atherosclerosis progression, it cannot determine the relative contribution of cell types, the underlying mechanism of activation of K_Ca_3.1 (e.g. calcium, second messenger, membrane trafficking), or the temporal aspect of activation. The relative contribution of K_Ca_3.1 among cell types will require cell-specific, inducible silencing/overexpression and, potentially, fate tracking to elucidate the responsible cell type(s). Lastly, K_Ca_3.1 has been shown to be expressed in mitochondria [71–73], and the potential role of mitochondrial versus sarcolemmal K_Ca_3.1 is currently unknown and cannot be overlooked as a potential contributor to the observed effects on atherosclerosis [74–84].

## Supplementary Material

Supplemental Material

## Data Availability

The data that support the findings of this study are available from the corresponding author, DKB, upon reasonable request.

## Highlights

1. Global genetic knockout of K_Ca_3.1 in mice results in smaller atherosclerotic plaques with a reduced necrotic core, resulting in a lesion with more stable plaque characteristics.
2. Silencing K_Ca_3.1 leads to a significant reduction in smooth muscle cell content within the plaques, consistent with a role for K_Ca_3.1 in smooth muscle proliferation and medial-to-intimal cell migration during atherosclerosis.
3. This study further demonstrates that K_Ca_3.1 regulates macrophage content in plaques implicating its inhibition in reducing macrophage infiltration and/or proliferation.
4. RNA sequencing predicted a novel K_Ca_3.1/cfos/PPAR axis, which may play a potential role in inhibiting atherosclerosis.
5. The study also shows K_Ca_3.1-mediated regulation of MCP1/Ccl2 upregulation in smooth muscle cells, not endothelial cells,
