# Supplementary material for: Genetic silencing of KCa3.1 inhibits atherosclerosis in ApoE null mice
Source: Channels (Austin). 2025 Aug 3;19(1):2538864. doi: 10.1080/19336950.2025.2538864 (PMC12320860; doi:10.1080/19336950.2025.2538864)
Supplement: Supplemental Material [file KCHL_A_2538864_SM5099.zip › Supplementary files/Alam_Supplementary_Table_1.docx]

***Supplementary Table 1:*** Up and down regulated genes in DKO associated with the IPA-identified top upstream regulator, i.e. the PPAR agonist, rosiglitazone. DKO resulting in differential expression in 45 of 52 predicted DEGs associated with rosiglitazone.

| **ID** | **Genes in dataset** | **Prediction (based on measurement direction)** | **Expr Log Ratio** | **Findings** |
| --- | --- | --- | --- | --- |
| 12869 | Cox8b | Activated | 3.264 | Upregulates (1) |
| 26458 | SLC27A2 | Inhibited | 3.074 | Downregulates (2) |
| 12683 | CIDEA | Activated | 3.01 | Upregulates (4) |
| 12865 | COX7A1 | Activated | 2.965 | Upregulates (1) |
| 22227 | UCP1 | Activated | 2.932 | Upregulates (21) |
| 18534 | PCK1 | Activated | 2.83 | Upregulates (4) |
| 22229 | UCP3 | Activated | 2.663 | Upregulates (3) |
| 66968 | PLIN5 | Activated | 2.586 | Upregulates (4) |
| 11537 | CFD | Activated | 2.534 | Upregulates (5) |
| 11812 | APOC1 | Activated | 2.515 | Upregulates (1) |
| 11450 | ADIPOQ | Activated | 2.505 | Upregulates (16) |
| 232493 | GYS2 | Activated | 2.452 | Upregulates (2) |
| 11832 | AQP7 | Activated | 2.386 | Upregulates (1) |
| 14311 | CIDEC | Activated | 2.329 | Upregulates (8) |
| 67800 | DGAT2 | Activated | 2.323 | Upregulates (2) |
| 11770 | FABP4 | Activated | 2.217 | Upregulates (44) |
| 243270 | HCAR1 | Activated | 1.934 | Upregulates (5) |
| 14555 | GPD1 | Activated | 1.916 | Upregulates (5) |
| 11556 | ADRB3 | Activated | 1.895 | Upregulates (1) |
| 14077 | FABP3 | Activated | 1.871 | Upregulates (4) |
| 52538 | ACAA2 | Activated | 1.73 | Upregulates (1) |
| 19013 | PPARA | Activated | 1.725 | Upregulates (2) |
| 16890 | LIPE | Activated | 1.641 | Upregulates (2) |
| 15439 | HP | Affected | 1.614 | Regulates (1) |
| 231086 | HADHB | Activated | 1.571 | Upregulates (2) |
| 12479 | CD1D | Activated | 1.556 | Upregulates (1) |
| 27273 | PDK4 | Activated | 1.455 | Upregulates (9) |
| 51798 | ECH1 | Activated | 1.388 | Upregulates (2) |
| 19016 | PPARG | Activated | 1.319 | Upregulates (28) |
| 11364 | ACADM | Activated | 1.298 | Upregulates (1) |
| 11363 | ACADL | Activated | 1.296 | Upregulates (1) |
| 13063 | CYCS | Activated | 1.26 | Upregulates (7) |
| 57875 | ANGPTL4 | Activated | 1.229 | Upregulates (1) |
| 15107 | HADH | Activated | 1.222 | Upregulates (2) |
| 11429 | ACO2 | Activated | 1.203 | Upregulates (1) |
| 22272 | UQCRQ | Activated | 1.177 | Upregulates (1) |
| 110842 | ETFA | Activated | 1.177 | Upregulates (2) |
| 12577 | CDKN1C | Affected | 1.129 | Regulates (1) |
| 12858 | COX5A | Activated | 1.127 | Upregulates (2) |
| 67680 | SDHB | Activated | 1.104 | Upregulates (1) |
| 30924 | ANGPTL3 | Affected | 1.102 | Regulates (1) |
| 11370 | ACADVL | Activated | 1.085 | Upregulates (1) |
| 12606 | CEBPA | Activated | 1.072 | Upregulates (4) |
| 12867 | COX7C | Activated | 1.072 | Upregulates (1) |
| 57279 | SLC25A20 | Activated | 1.064 | Upregulates (2) |
| 12859 | COX5B | Activated | 1.055 | Upregulates (2) |
| 16922 | PHYH | Activated | 1.03 | Upregulates (1) |
| 12491 | CD36 | Activated | 1.023 | Upregulates (25) |
| 70673 | PRDM16 | Inhibited | -1.103 | Upregulates (2) |
| 14281 | FOS | Inhibited | -1.425 | Upregulates (1) |
| *© 2000-2024 QIAGEN. All rights reserved.* | | | | |
