# Supplementary figures and images for: Genetic silencing of KCa3.1 inhibits atherosclerosis in ApoE null mice
Source: Channels (Austin). 2025 Aug 3;19(1):2538864. doi: 10.1080/19336950.2025.2538864 (PMC12320860; doi:10.1080/19336950.2025.2538864)

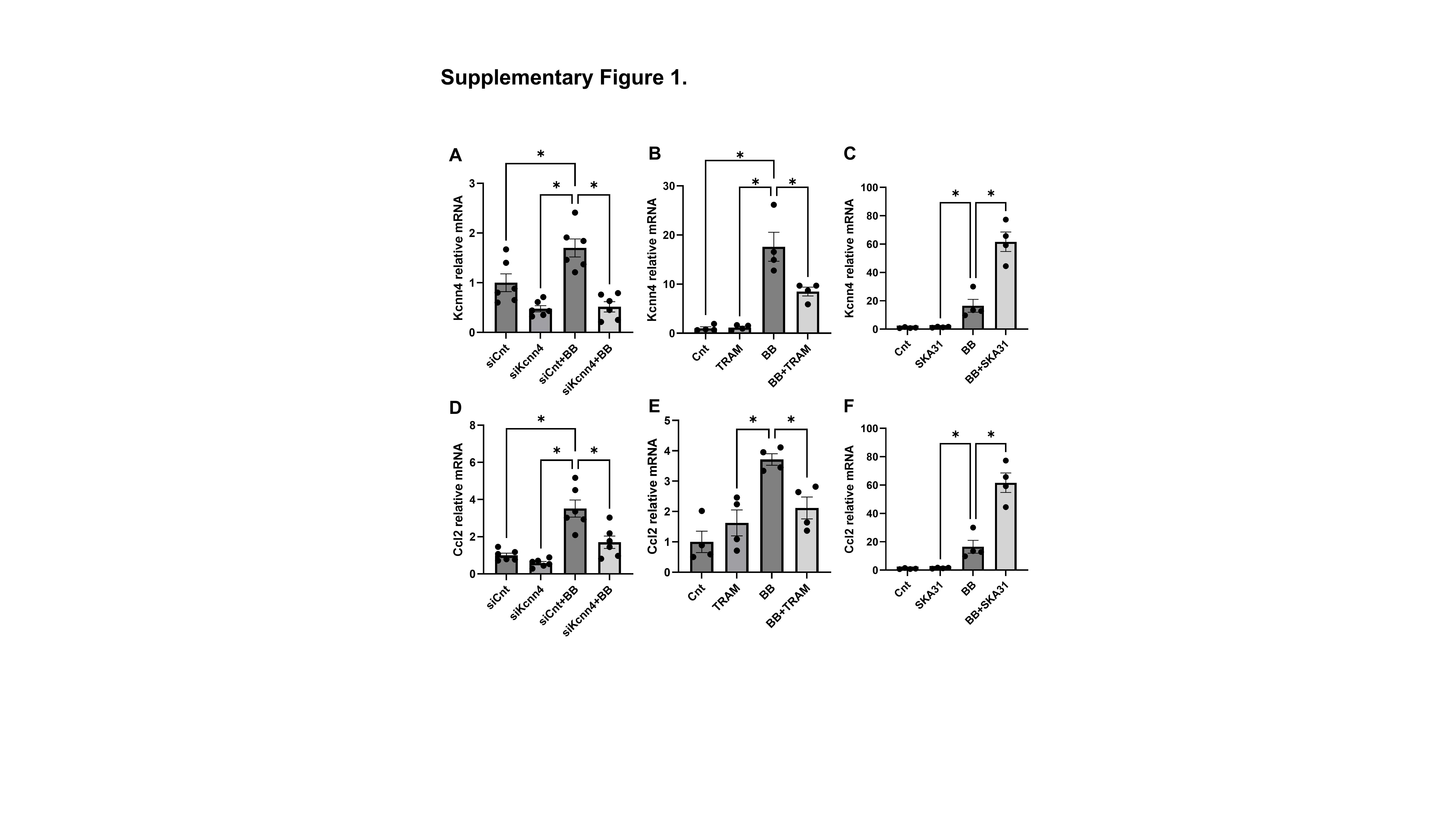

Supplement: Supplemental Material [file KCHL_A_2538864_SM5099.zip › Supplementary files/Supplementary_Figure_1.JPG]

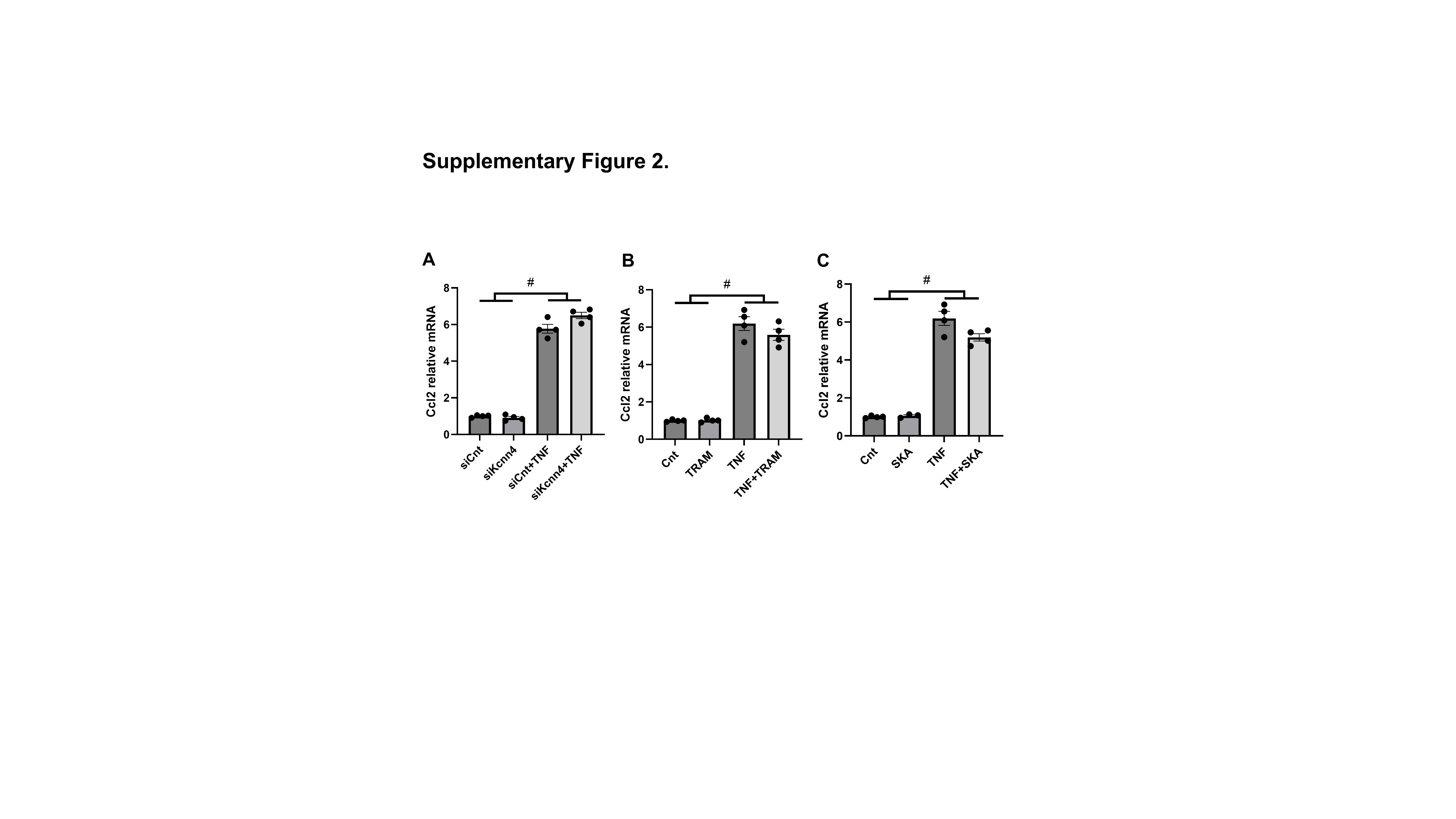

Supplement: Supplemental Material [file KCHL_A_2538864_SM5099.zip › Supplementary files/Supplementary_Figure_2.JPG]

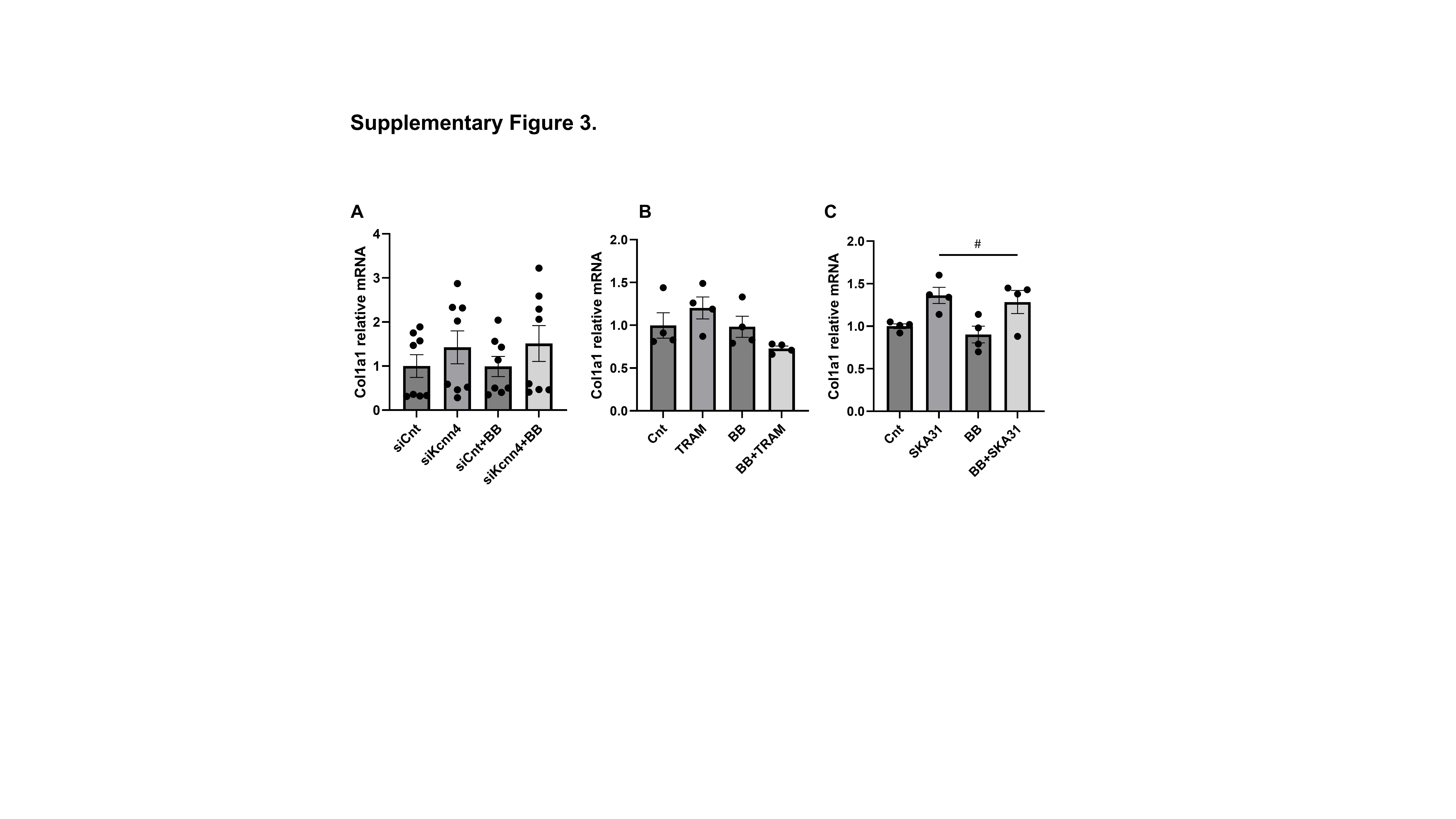

Supplement: Supplemental Material [file KCHL_A_2538864_SM5099.zip › Supplementary files/Supplementary_Figure_3.JPG]

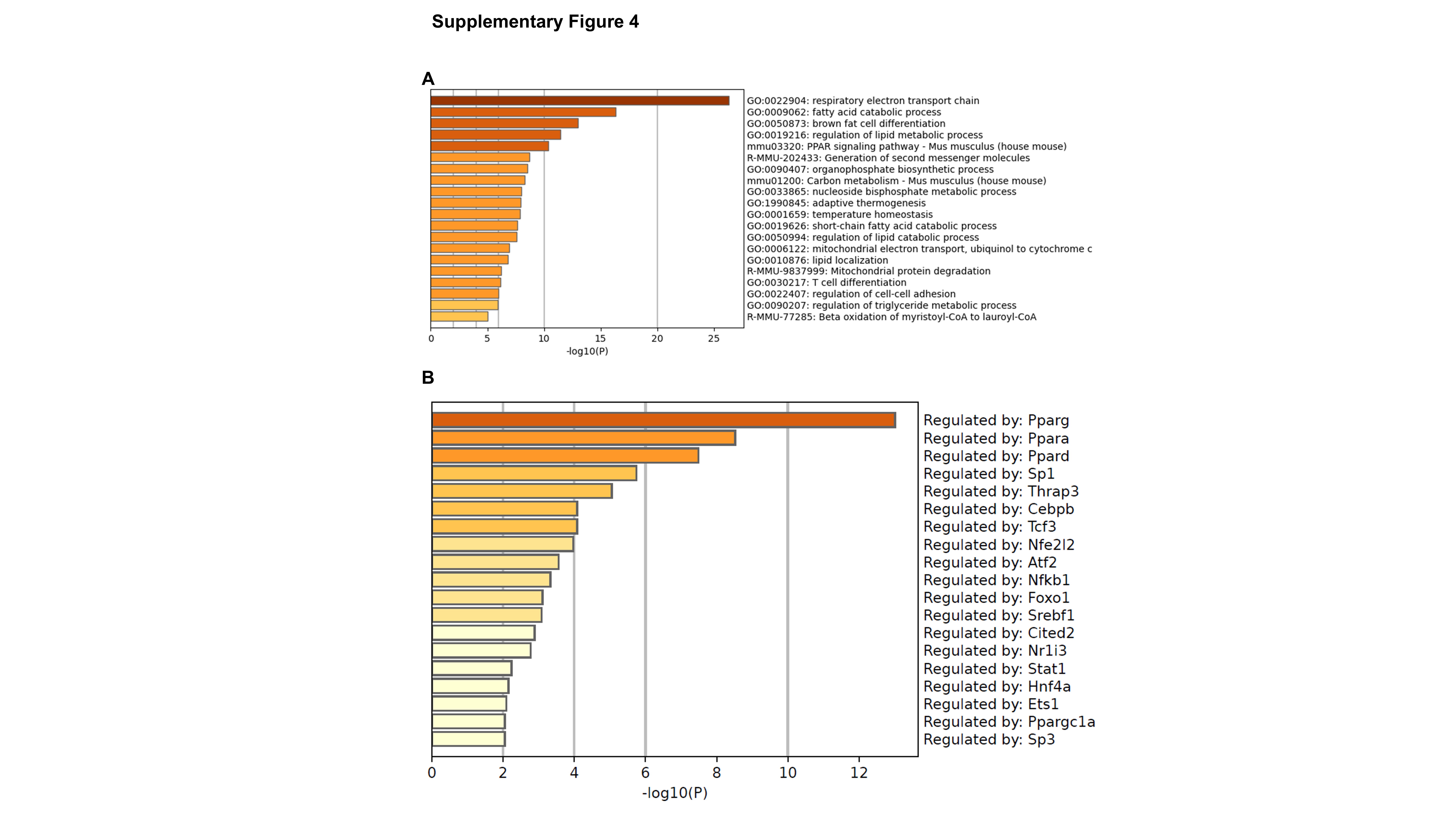

Supplement: Supplemental Material [file KCHL_A_2538864_SM5099.zip › Supplementary files/Supplementary_Figure_4.JPG]
